# Effect of graded posterior element and ligament removal on annulus stress and segmental stability in lumbar spine stenosis: a finite element analysis study

**DOI:** 10.3389/fbioe.2023.1237702

**Published:** 2023-09-18

**Authors:** Maohua Lin, James Doulgeris, Utpal Kanti Dhar, Timothy O’Corner, Ioannis Dimitri Papanastassiou, Chi-Tay Tsai, Frank D. Vrionis

**Affiliations:** ^1^ Department of Ocean and Mechanical Engineering, Florida Atlantic University, Boca Raton, FL, United States; ^2^ Department of Neurosurgery, Marcus Neuroscience Institute, Boca Raton Regional Hospital, Boca Raton, FL, United States; ^3^ Department of Orthopedic, General Oncological Hospital Kifisias “Agioi Anargryroi”, Athens, Greece

**Keywords:** unilateral laminotomy, complete laminectomy, facetectomy, shear stress, von-Mises stress, annulus fiber, finite element, ROM

## Abstract

The study aimed to investigate the impact of posterior element and ligament removal on the maximum von Mises stress, and maximum shear stress of the eight-layer annulus for treating stenosis at the L3-L4 and L4-L5 levels in the lumbar spine. Previous studies have indicated that laminectomy alone can result in segmental instability unless fusion is performed. However, no direct correlations have been established regarding the impact of posterior and ligament removal. To address this gap, four models were developed: Model 1 represented the intact L2-L5 model, while model 2 involved a unilateral laminotomy involving the removal of a section of the L4 inferior lamina and 50% of the ligament flavum between L4 and L5. Model 3 consisted of a complete laminectomy, which included the removal of the spinous process and lamina of L4, as well as the relevant connecting ligaments between L3-L4 and L4-L5 (ligament flavum, interspinous ligament, supraspinous ligament). In the fourth model, a complete laminectomy with 50% facetectomy was conducted. This involved the same removals as in model 3, along with a 50% removal of the inferior/superior facets of L4 and a 50% removal of the facet capsular ligaments between L3-L4 and L4-L5. The results indicated a significant change in the range of motion (ROM) at the L3-L4 and L4-L5 levels during flexion and torque situations, but no significant change during extension and bending simulation. The ROM increased by 10% from model 1 and 2 to model 3, and by 20% to model 4 during flexion simulation. The maximum shear stress and maximum von-Mises stress of the annulus and nucleus at the L3-L4 levels exhibited the greatest increase during flexion. In all eight layers of the annulus, there was an observed increase in both the maximum shear stress and maximum von-Mises stress from model 1&2 to model 3 and model 4, with the highest rate of increase noted in layers 7&8. These findings suggest that graded posterior element and ligament removal have a notable impact on stress distribution and range of motion in the lumbar spine, particularly during flexion.

## Introduction

Lumbar spinal stenosis, which refers to the narrowing of the spinal canal in the lumbar region, is a prevalent spinal disorder in older individuals (Szpalski and Gunzburg, 2003). This condition is often caused by the degeneration and overgrowth of joints between the vertebral segments, leading to nerve root compression and subsequent lower back and leg pain (Arbit et al., 2001). For individuals over 65, lumbar spinal stenosis is a primary reason for spinal surgery (Lurie and Tomkins-Lane, 2016). Thus, surgical intervention typically involves the extensive removal of posterior spinal structures, including the interspinous ligaments, spinous processes, bilateral laminae, portions of the facet joints and joint capsules, and the ligamentum flavum (Guiot et al., 2002; Bresnahan et al., 2009).

Unilateral laminotomy, complete laminectomy, and facetectomy are three surgical techniques commonly employed to address conditions affecting the lumbar spine, particularly lumbar spinal stenosis (Epstein, 1995; Thomé et al., 2005). These procedures involve the removal of varying degrees of posterior elements, such as lamina, spinous processes, and facets. Posterior element removal, such as laminectomy or laminotomy, alters the load-bearing capacity of the spine by modifying the structures that contribute to spinal stability (Bresnahan et al., 2009). Posterior element removal might increase the flexibility of the spine, affecting its load-bearing capacity and potentially leading to adjacent segment degeneration (Kim et al., 2015). Ligament removal might alter load distribution, leading to increased stress on certain spinal structures and potentially contributing to degeneration (Iorio et al., 2016). Ligament removal, on the other hand, can lead to changes in load transmission and distribution across the vertebral segments (Goel et al., 2005). The biomechanical implications of these techniques, including posterior element removal and ligament removal, are of paramount importance in understanding their effects on spinal stability, load distribution, and patient outcomes.

Finite Element Analysis (FEA) is a commonly used method for investigating the biomechanics of the human lumbar spine (Eberlein et al., 2004; Dreischarf et al., 2014; Pawlikowski et al., 2015; Xu et al., 2017; Zhang et al., 2018). FEA allows researchers and clinicians to gain insights into the biomechanical behavior of the spine affected by stenosis. The von Mises stress is a measure of the equivalent stress experienced by a material under complex loading conditions (Piovesan et al., 2019). It provides a criterion to assess whether a material will undergo plastic deformation or failure. In the context of stenosis, the von Mises stress helps evaluate the structural integrity of the vertebra and identify regions prone to failure. Shear stress is another parameter of interest in FEA of stenosis. Maitirouzi et al. (2022) used von Mises stress and maximum von Mises stress criteria to evaluate the modified CBT (cortical bone trajectory) screw technique model, demonstrating improved mechanical stability. Furthermore, shear stress represents the force parallel to the surface per unit area. It is relevant because the pars interarticularis is subjected to significant shear forces during various activities, such as bending, twisting, or repetitive loading (Chang et al., 2022). High shear stresses can contribute to the development or progression of spondylolysis by causing microdamage and fatigue failure (Kaeding and Najarian, 2010). By performing FEA on a model of the spine, researchers can analyze the distribution of von Mises stress and shear stress. This information helps in understanding the biomechanical factors contributing to the development and progression of stenosis. It can guide treatment strategies, such as recommending modifications in activities or designing interventions like bracing or surgical procedures to reduce stress concentrations and enhance the structural integrity of the affected region.

In our previous works, we successfully developed a complex 3D FEA model in the cervical spine (Lin et al., 2021; Lin et al., 2022a; Lin et al., 2022b; Lin et al., 2023), thereby confirming the accurate representation of ligaments, nucleus pulposus, and annulus through solid modeling. In this study, we extended our model to the lumbar spine and focused on a section of the lumbar spine and did a comparative FEA on common treatments for spinal stenosis. These methodologies were aimed to provide insight into the mechanical response to treatments and determine if more investigations were warranted. We then evaluated the impact of posterior element and ligament removal on the ROM, pressure, maximum von Mises stress, and maximum shear stress of the nucleus and annulus for simulating treatment stenosis paradigms at the L3-L4 and L4-L5 levels in the lumbar spine.

## Experimental methods

### Model

**FIGURE 1:**
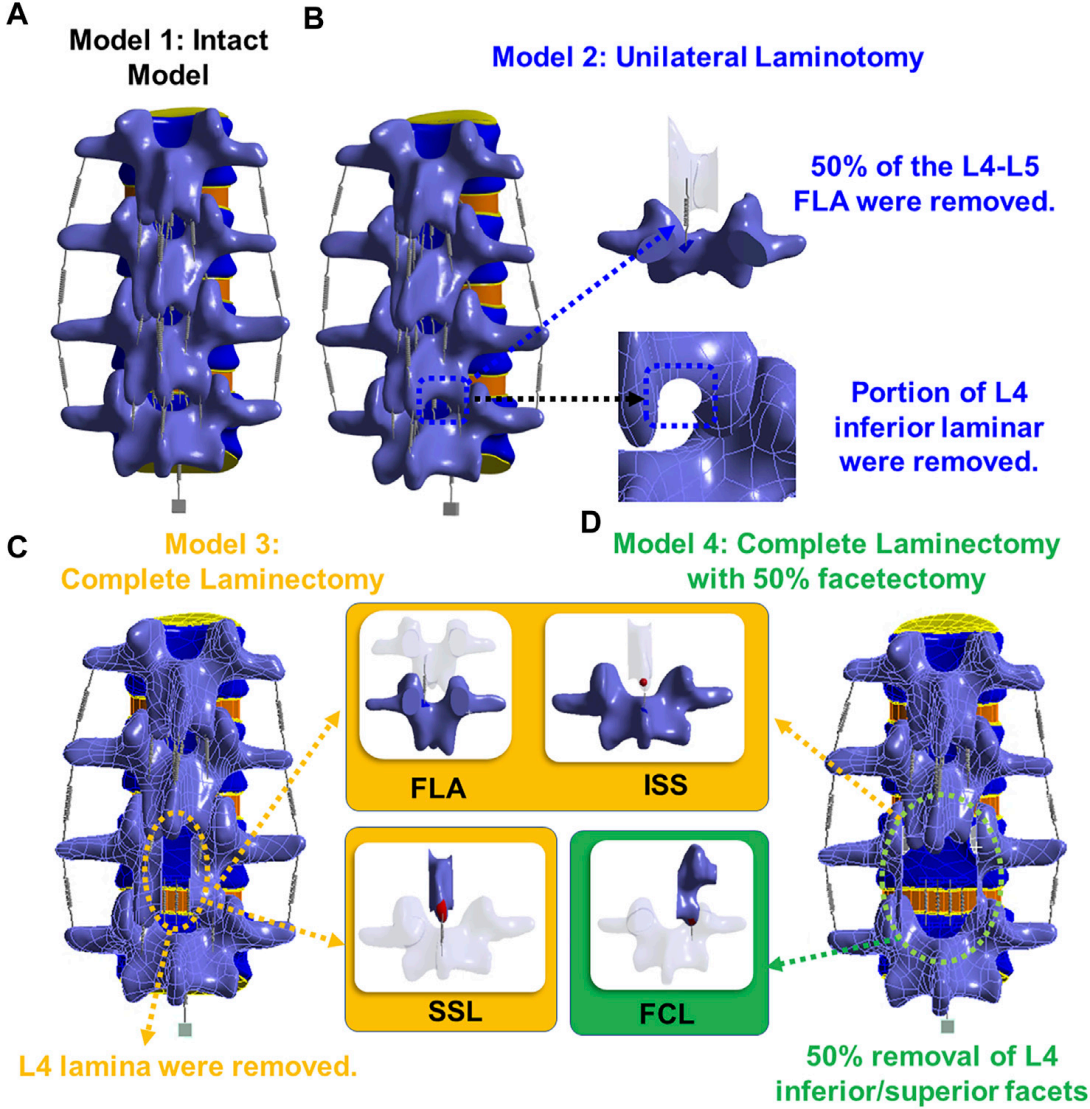
**(A)** Model 1: an intact model; **(B)** Model 2: unilateral laminotomy, where a portion of the L4 inferior lamina and 50% of the L4-L5 ligament flavum were removed; **(C)** Model 3: a complete laminectomy with the following removals: L4 spinous process, L4 lamina, and the relevant connecting ligaments of L3-L4 and L4-L5 (ligament flavum, interspinous ligament, supraspinous ligament); **(D)** Model 4: a complete laminectomy with 50% facetectomy with the following removals: the same removals from model three, 50% removal of L4 inferior/superior facets and 50% removal of the facet capsular ligaments of L3-L4 and L4-L5.

**FIGURE 2:**
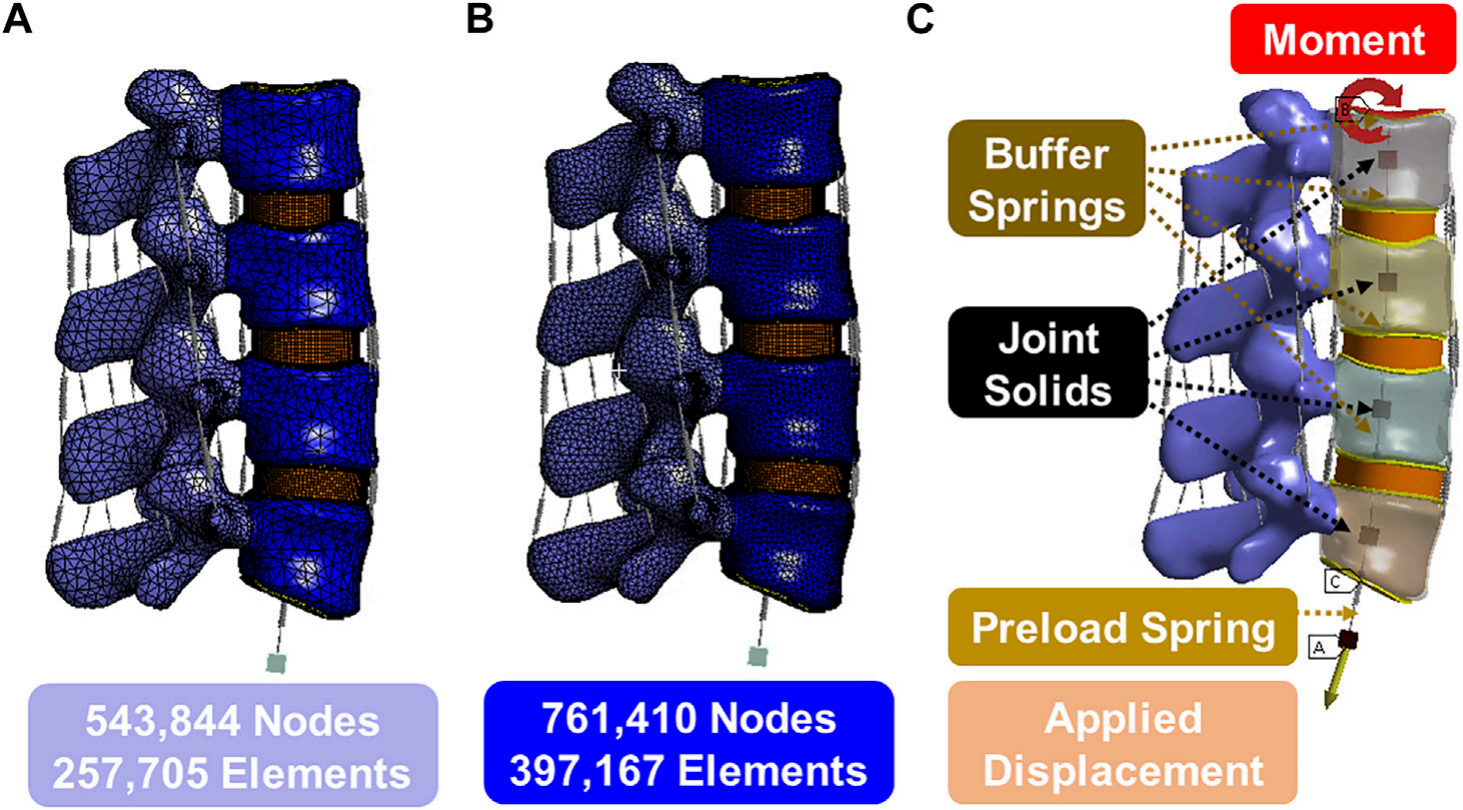
**(A)** Model 1 with 543,844 nodes and 257,705 elements; **(B)** Model 1 with 761,410 nodes 397,167 elements; **(C)** Boundary conditions.

**FIGURE 3:**
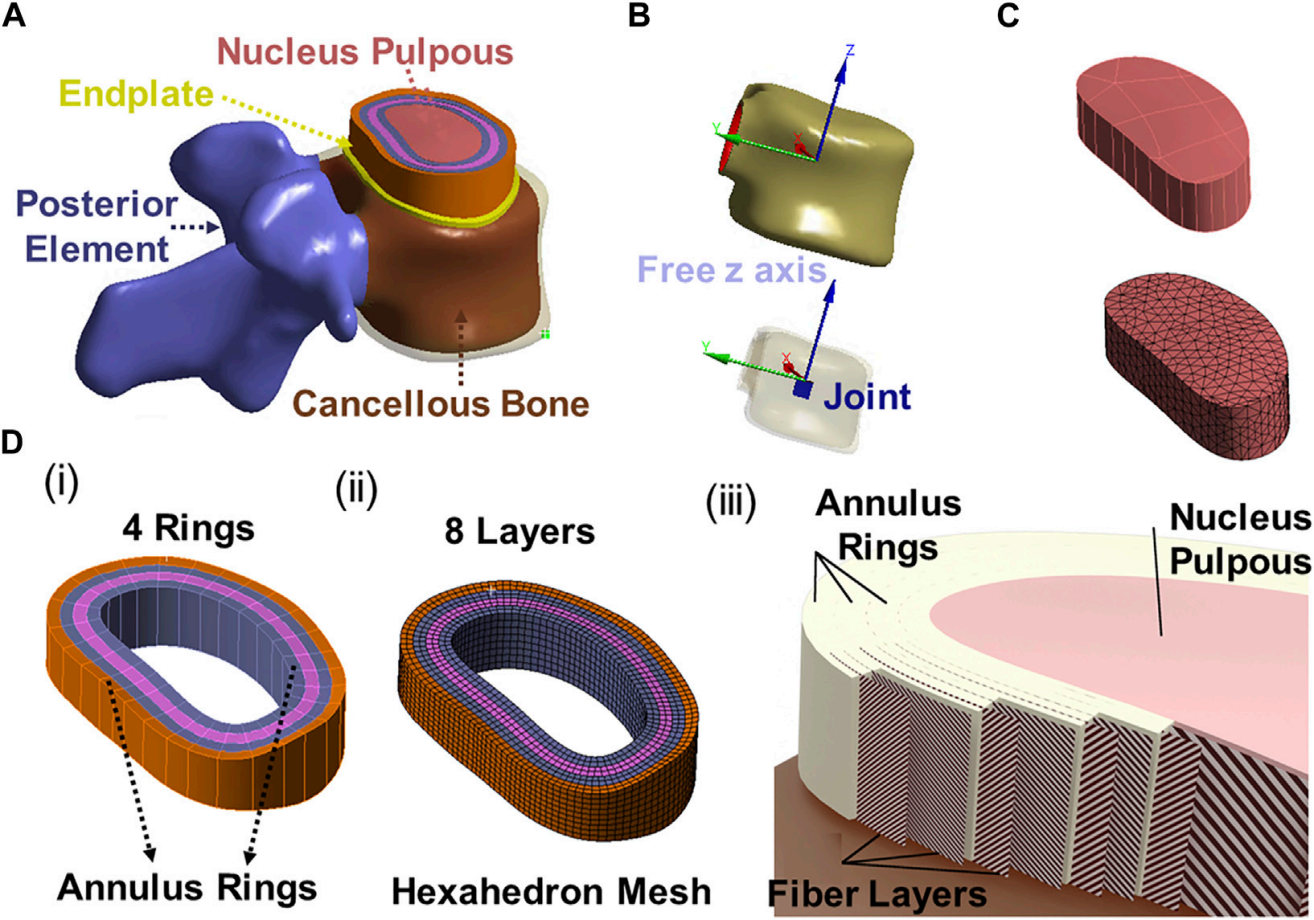
**(A)** 3D modeling of endplate, nucleus pulpous, posterior element and annulus; **(B)** The joint solids are connected to the inner surface of the cortical shell and are constrained in all degrees of freedom except the z direction (superior/inferior) relative to the top of the solid; **(C)** Nucleus geometry and mesh; **(D)** (i) 4 annulus rings, (ii) 8 annulus layer with hexahedron element, (iii) Fibers in annulus were in alternating ±30° orientations in one layer (inner fiber direction and outer fiber direction).

Lumbar spine CT images, at 0.75 mm slices, were obtained from a 62-year-old female patient with no spinal pathologies or anatomical anomalies. The CT was then imported into the Mimics program (Materialise, Leuven, Belgium) and a shell of the lumbar (L2-L5) vertebral body was generated. These shells were smoothed by software and any unusual bony protrusions were removed. The Mimics software generated geometric solids of the cortical shell and cancellous core (Figures 1–3). Statistics show that the average thickness of the anterior cortical shell was 0.75 ± 0.125 mm (Palepu et al., 2019). The generated cortical shell and cancellous core were integrated into Solidworks software (Dassault Systèmes, France) for preprocessing and generation of other soft tissue 3D models. The posterior elements were separated from the shell/core solid bodies and merged into a single solid. The endplates were created on the superior/inferior surfaces of the vertebral body and were 0.75 mm thick. The nucleus pulposus was added at the center of the upper and lower endplates adequately, and four 1.5 mm thick concentric rings were added around the nucleus pulposus to form the annulus fibrosus. The ellipsoid configuration of the annulus fibrosis and nucleus pulposus was adopted from the research conducted by Shirazi-Adl (Shirazi-Adl et al., 1986) and Eiberlein (Eberlein et al., 2004). This ellipsoidal shape has been widely utilized in finite element studies. The dimensions of the ellipsoid were determined by considering the morphology of the vertebral body, with the maximal area being selected. Applying this approach to the analyzed specimens yielded disc volumes ranging from 5 to 15 cm³, contingent upon factors such as disc height and gender. These outcomes align well with documented literature values, as observed in the work by Malko (Malko et al., 2002). Cubic solids were added to the center of the vertebral bodies, which were used for preloaded and simulated muscle connections.

The 3D model generated by Solidworks was imported into ANSYS R21 for preprocessing. Nonlinear tension spring elements were used in the model (Model 1) and these springs were added to each spinal level to simulate the lumbar ligaments; anterior longitudinal ligament (ALL), posterior longitudinal ligament (PLL), transverse ligament (TL), ligamentum flavum (FLA), interspinous ligament (ISL), supraspinous ligament (SSL) and facet capsular ligament (FCL). The annulus was composed of 4 rings (Figure 3Di), and each ring is divided into two layers (Figure 3DiiD). Reinforcement tension-only fibers were embedded in the annulus ring bodies at 25% and 75% locations from the other most surface of each annulus ring, alternating in ±30° directions (Figure 3Diii). The material properties of these fibers were obtained from the works of Shirazi-Adl et al. The fiber strength decreased as it moved from the outermost layer to the innermost layer, as shown in Table 1 (Shirazi-Adl et al., 1986). Tetrahedron elements were used for all solid bodies excluding the annulus where hexahedron elements were used. A convergence analysis confirmed that the mesh sizing was sufficient with a convergence criterion of less than 5%. The solution under the convergence test did not change significantly with mesh refinement between the final two models (shown in Figures 2A, B). With meshing refinement, the solution using a convergence test did not change appreciably between model 1 with 543,844 nodes and 257,705 elements and model 1 with 761,410 nodes 397,167 elements.

**TABLE 1:** Summary of material boundary conditions used in this analysis.

Component	Material properties	References
Cortical Bone	E = 12,000 MPa	Xu et al. (2017)
	ν = 0.30	
Posterior Elements	E = 3,500 MPa	Xu et al. (2017)
	ν = 0.30	
Cancellous Bone	E = 100 MPa	Xu et al. (2017)
Endplates	E = 23.8 MPa	Xu et al. (2017)
	ν = 0.40	
Annulus ground substance	Hyperelastic c_1_ = 0.56, c_2_ = 0.14	Schmidt et al. (2006)
Nucleus Pulpous	Hyperelastic c_1_ = 0.12, c_2_ = 0.09	Schmidt et al. (2006)
Ligaments	Nonlinear stress-strain curves, tension only	Averaged values between Eberlein et al., 2004; Shirazi-Adl et al., 1986
ALL	CSA = 35 mm^2^	
PLL	CSA = 15 mm^2^	
FLA	CSA = 75 mm^2^	
FCL	CSA = 50 mm^2^	
TL	CSA = 8 mm^2^	
ISL	CSA = 35 mm^2^	
SSL	CSA = 30 mm^2^	
Annulus fiber	Nonlinear stress-strain curves, tension only	Shirazi-Adl et al. (1986)
Layer 1 and 2 (Inner most Layer)	Elasticity ratio = 0.65	
	CSA = .20 mm^2^	
Layer 3 and 4	Elasticity ratio = 0.75	
	CSA = .20 mm^2^	
Layer 5 and 6	Elasticity ratio = 0.90	
	CSA = .20 mm^2^	
Layer 7 and 8 (Outer most layer)	Elasticity ratio = 1.00	
	CSA = .20 mm^2^	

This study considered four different models shown in Figure 3. The first model was an intact specimen for baseline comparisons. The second model was a unilateral laminotomy, where a portion of the L4 inferior lamina and 50% of the L4-L5 ligament flavum were removed. The third model was a complete laminectomy with the following removals: L4 spinous process, L4 lamina, and the relevant connecting ligaments of L3-L4 and L4-L5 (ligament flavum, interspinous ligament, supraspinous ligament). The fourth model was a complete laminectomy with 50% facetectomy with the following removals: the same removals from model three, 50% removal of L4 inferior/superior facets and 50% removal of the facet capsular ligaments of L3-L4 and L4-L5.

### Material characteristics

**FIGURE 4:**
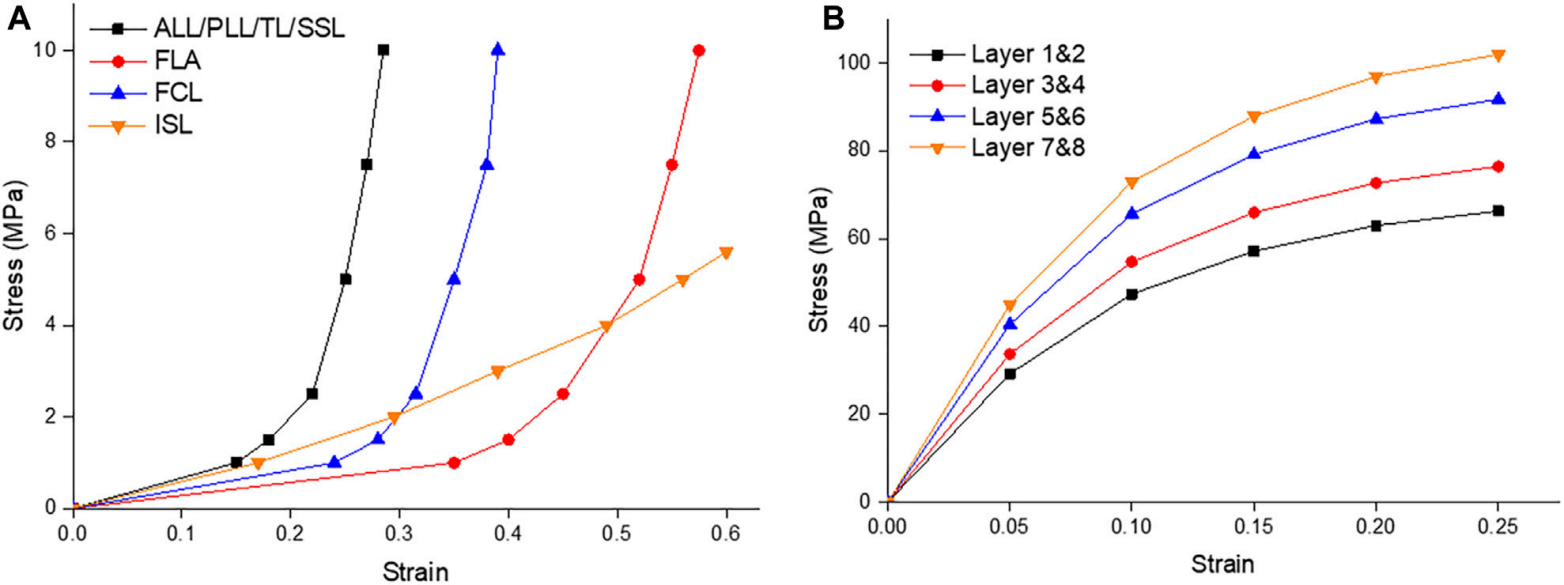
**(A)** Ligament stress strain curves of the Anterior Longitudinal Ligament (ALL), Posterior Longitudinal Ligament (PLL), Transverse Ligament (TL), Supraspinous Ligament (SSL), Ligament Flavum (FLA), Facet Capsular Ligament (FCL), and Interspinous Ligament (ISL); **(B)** The fiber stress strain curves of the innermost layers (1 & 2) to the outermost layers (7 & 8) (Shirazi-Adl et al., 1986; Eberlein et al., 2004).

Table 1 summarizes the material parameters of this study (Shirazi-Adl et al., 1986; Eberlein et al., 2004; Schmidt et al., 2006; Xu et al., 2017). The ligament spring properties were derived from the stress-strain values of Eberlein 2004 to create the values for these ligaments and were summarized in Table 1; Figure 4A. Fiber material properties were derived from the work of Shirazi-Adl et al. (1986). The fibers in the outermost layer have the highest elastic strength, and each concentric layer decreases by a scalar factor as it reaches the innermost layer, as shown in Table 1; Figure 4B.

### Boundary, contact and loading conditions

The lower surface of the L5 endplate is constrained. The upper surface of the L2 endplate is not constrained but serves as a location for moment application. A linear spring, that cannot exceed a pre-determined max load, is attached to the upper surface of the L2 endplate to the floating rigid body to simulate the loads from muscles and torso weight. Figure 2C illustrates the follower preload application boundary condition visually. It depicts four joint solids (center of the gravity), one displacement solid, four buffer springs, and one preload spring. The joint solids are connected to the inner surface of the cortical shell via a join command and are constrained in all degrees of freedom except the z direction (superior/inferior) relative to the top of the solid. The buffer springs and preload spring are attached to the joint solids and allow only rotational degrees of freedom, which mimics the physiological loading conditions experienced by the spine in an upright posture. This hypothesis is grounded in the understanding that the spine’s load-bearing capacity and response to external forces are influenced by its natural physiological alignment and load distribution. The buffer springs have significantly higher stiffness and maximum load compared to the preload spring, enabling force transmission without rigid connection. The preload spring contains a max load command that will be reached after 5 mm of deformation and will stay at that max load (determined by the preload value) regardless of additional displacement. The bottommost solid is displaced to stretch the preload spring and apply the desired force to the top of the system. The joints function as simulated muscles to regulate the preload and prevent excessive rotation during the preload application. This approach is commonly known as a follower preload, as the applied force aligns with the normal vector of the top endplate regardless of rotation.

Moment and follower preload values depend on the expected motion as follows: 7.5Nm and 1175N for flexion, 7.5Nm and 500N for extension, 7.8Nm and 700N for transverse bending, and 5.5Nm and 720N for axial rotation. These loading conditions were selected from the works of Dreischarf et al. (2014) and Rohlmann et al. (2009), but the preloading method was based on Rohlmann’s analysis of the loading method. The loading example consists of 2 steps: step 1 ramps the preload to a maximum value over 60 s, and step 2 ramps the applied torque to a maximum value over 120 s. All soft and hard tissue connections are computed using the adhesive contact method with symmetric (target and contact) detection. The facet joint of the posterior element was performed using the frictional contact method (µ = 0.05) and a 0.75 mm pinball area assuming initial contact. All the other contacts, including the vertebrae and intervertebral discs, were assumed to be bonded. Meshing included patch-independent body sizing for all bodies except the annulus, which used a multi-region approach and mapped face meshing. The latter is required to ensure that the fiber reinforcement codes are correctly added to the model in the correct orientation and position. All fiber reinforcements are verified by exporting vector images combined with human review before the first solution.

The finite element model will be validated through a comprehensive process that involves comparing the simulated results with existing experimental data and relevant findings from the literature. The validation will be focused on assessing the accuracy of the model’s responses under different loading conditions, encompassing flexion, extension, lateral bending, and axial rotation. ROM and intradiscal pressures will be compared between the model’s predictions and literature values for different loading scenarios. The model’s results will be checked to see if they fell within the reported ranges. To ensure a comprehensive validation, the loading conditions applied in the simulations will be matched to those used in previous experimental studies. This approach allowed for a direct comparison between the model’s responses and the real-world biomechanical measurements, enhancing the credibility of the validation process.

## Results

### Model validation

**FIGURE 5:**
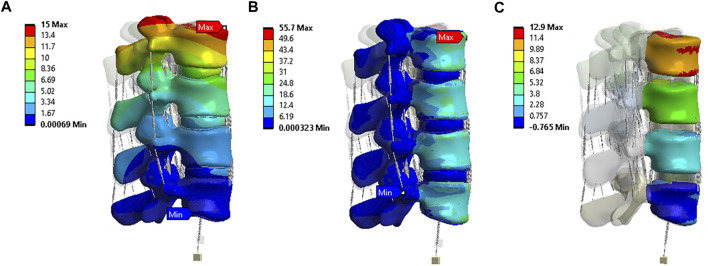
Flexion situation results under model 1: **(A)** Displacement (mm); **(B)** von-Mises stress (MPa); **(C)** Rotation (^0^).

**FIGURE 6:**
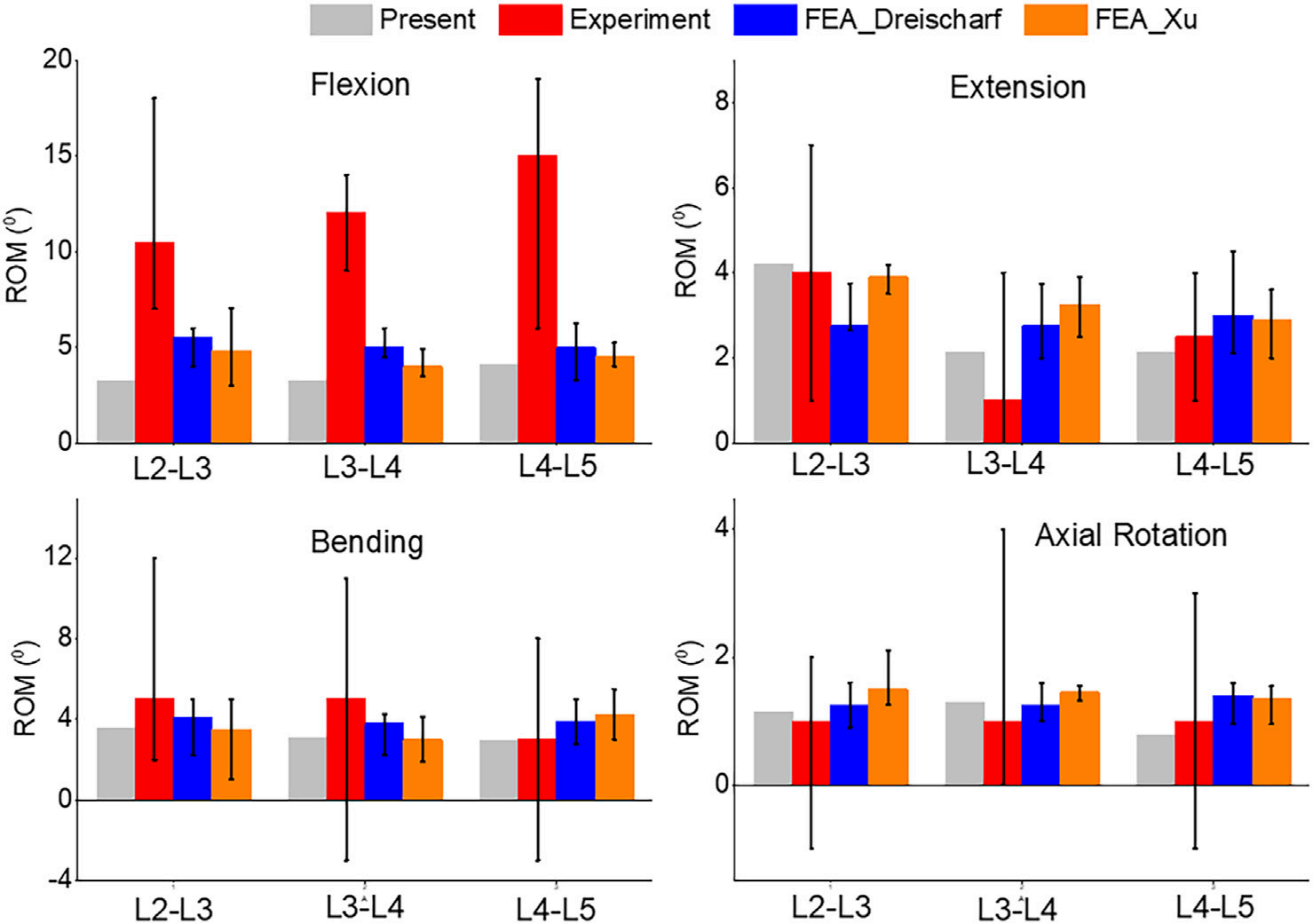
Comparison of median and range of intervertebral rotations (top to bottom) of Flexion, Extension, Lateral Bending, and Axial Rotation from the current study, *in vivo* results (Pearcy, 1985), and FEA from the literature (Dreischarf et al., 2014; Xu et al., 2017).

To validate the accuracy of the finite element (FE) models, the range of motion (ROM) was compared with previous experimental (*in vivo* or *in vitro*) and FE studies. Figure 5 shows the displacement, von-Mises stress, and rotation of model 1 (intact model) of the L2-L5 lumbar spine during flexion. The segmental ROM for model 1 corresponded to the range of reported results for all modes of motion. The results were consistent with the range of other FEA models and *in vivo* studies for all movements (Figure 6). The model had consistently lower median values in flexion and, similar to the FEA literature, exceeded the *in vivo* range at some spinal levels.

**FIGURE 7:**
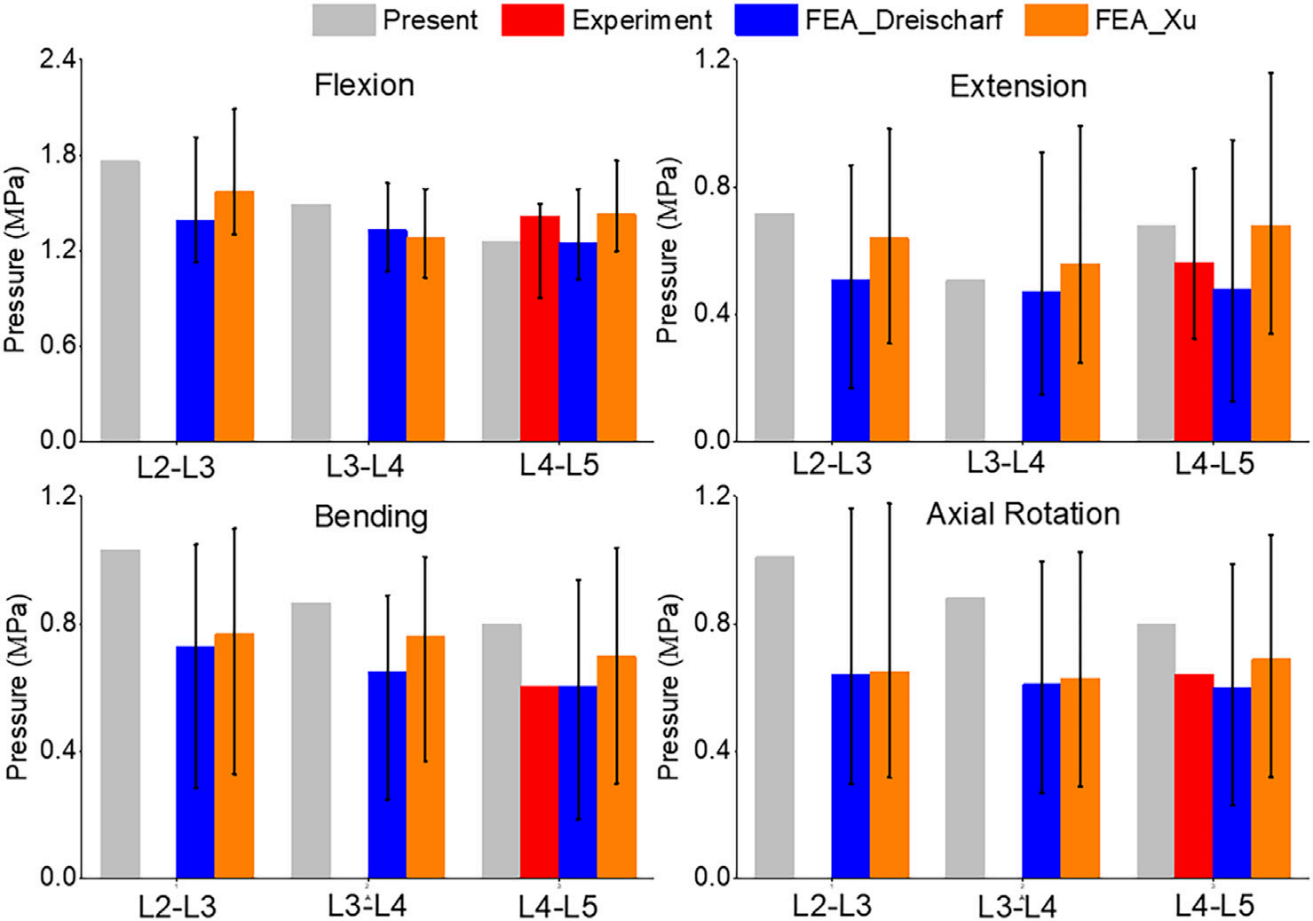
Comparison of median and range of intra discal pressure (top to bottom) of Flexion, Extension, Lateral Bending, and Axial Rotation from the current study, *in vivo* results (Sato et al., 1999; Wilke et al., 2001), and FEA from the literature (Dreischarf et al., 2014; Xu et al., 2017).

A comparison of the pressures can be seen in Figure 7. Flexion and extension intradiscal pressures are consistent with *in vivo* and literature at all spinal levels. This study produced higher intradiscal pressures during lateral flexion and axial rotation, but the results were within the range of the literature. However, intradiscal pressures were higher than the single *in vivo* measurement at the L5 disc in lateral bending and axial rotation.

### ROM

**FIGURE 8:**
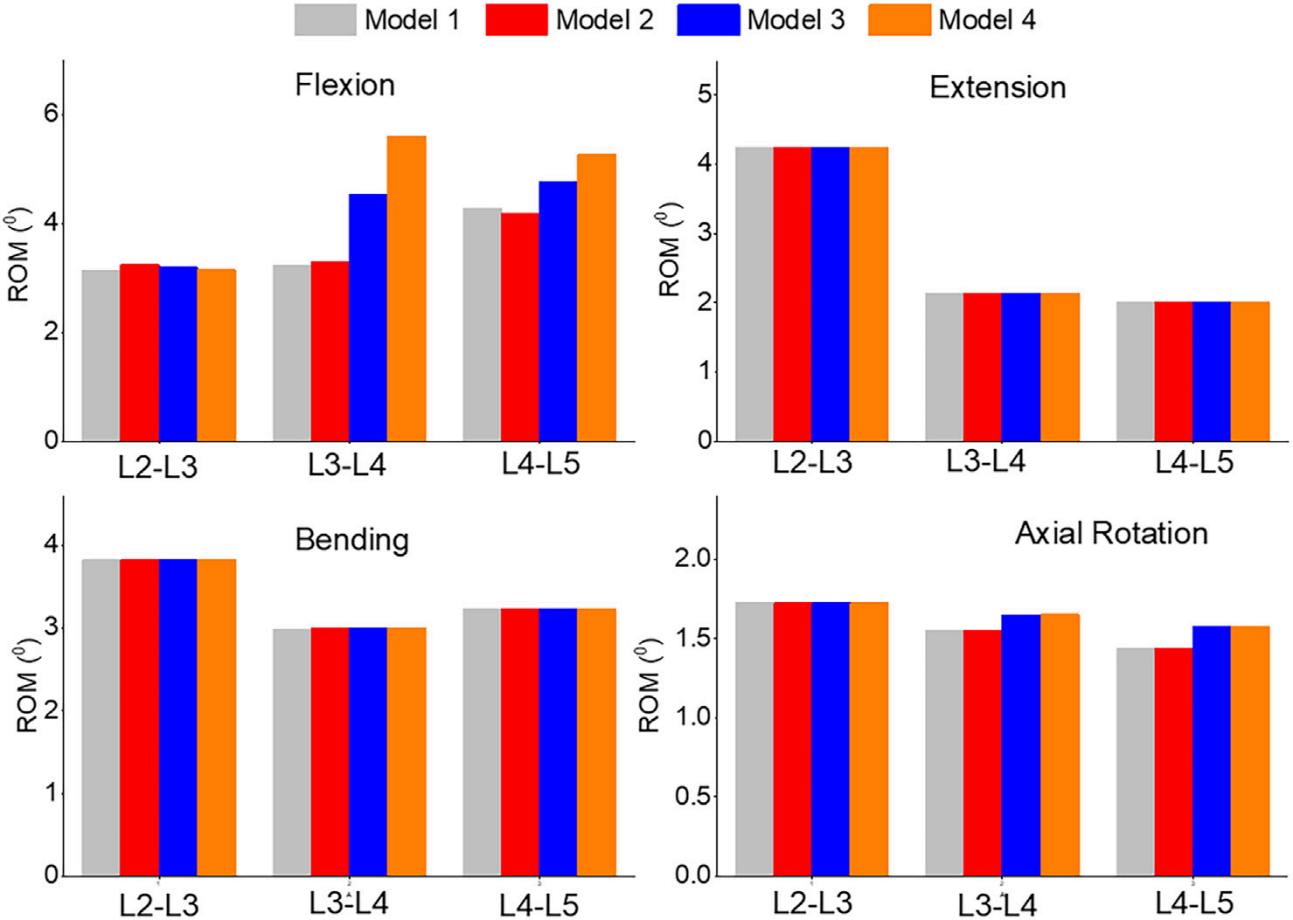
Comparison of ROM of Flexion, Extension, Lateral Bending, and Axial Rotation in all four models.

In Figure 8, the range of motion (ROM) for flexion, extension, bending, and axial rotation was compared across all four models. During the flexion simulation, the ROM in L3-L4 showed the greatest increase, rising from 3.2° in model 1 and 2 to 4.5° in model 3 and 5.6° in model 4. The ROM in L4-L5 also increased by 10% from model 1 and 2 to model 3, and by 20% to model 4. Unilateral laminotomy involves the removal of a portion of the lamina and the underlying ligamentum flavum unilaterally, while preserving the contralateral structures. Ligament removal is limited to the ligamentum flavum on the side of the laminotomy. Complete laminectomy entails the complete removal of the lamina and spinous processes, which results in increased spinal flexibility and altered load distribution across the vertebral segments due to the extensive posterior element removal. Facetectomy involves the removal of the facet joint, which contributes to neural foraminal decompression and alleviation of nerve root compression. Facetectomy significantly affects the stability and load-bearing capacity of the spine. The loss of the facet joint diminishes the constraint on rotational movements, potentially leading to increased segmental motion.

### Pressure

**FIGURE 9:**
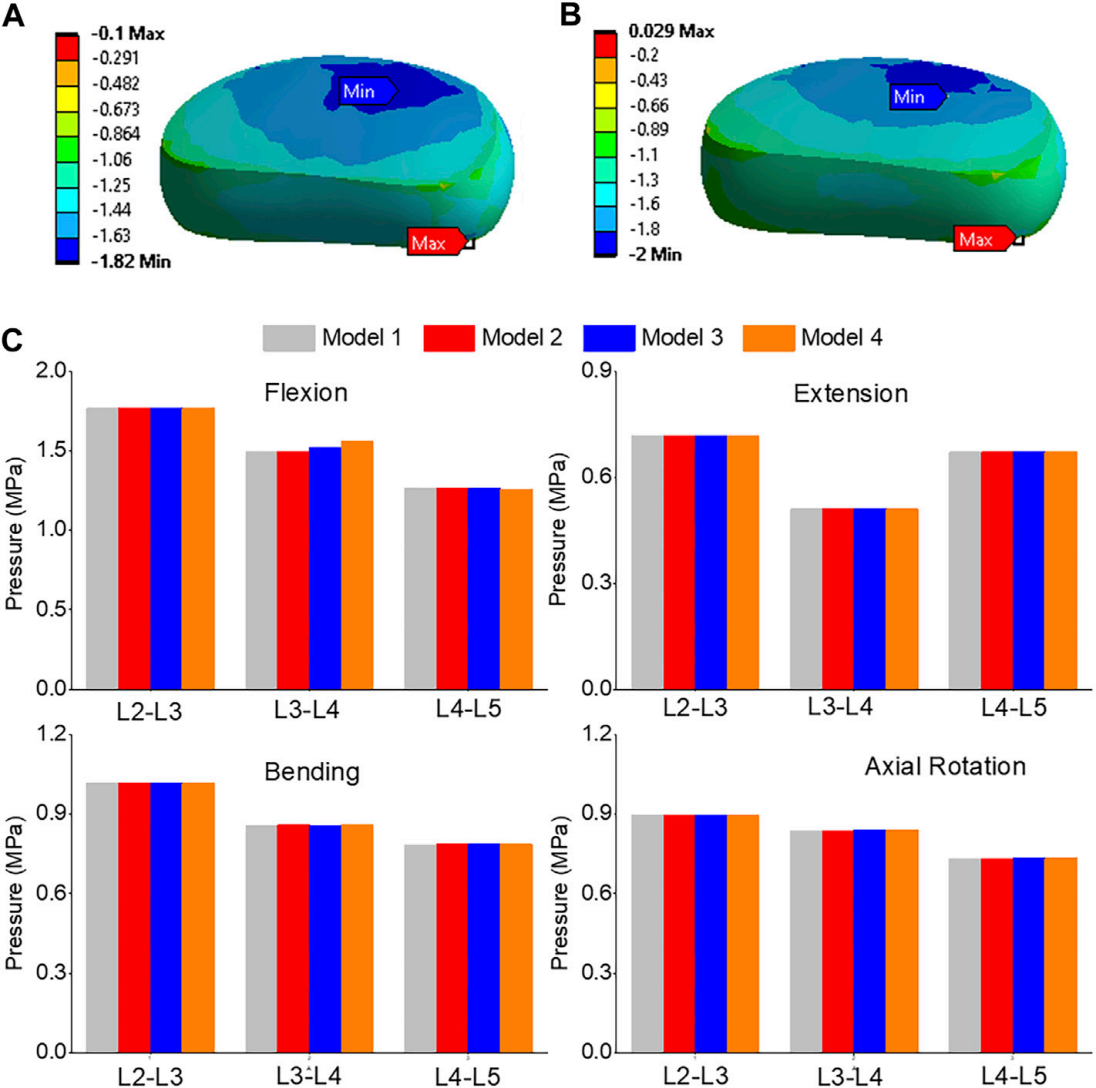
**(A)** A typical pressure contour of the L3-L4 under flexion for model 1; **(B)** A typical pressure contour of the L3-L4 under flexion for model 4; **(C)** Comparison of nucleus pressure of Flexion, Extension, Lateral Bending, and Axial Rotation in all four models.

The pressure distribution in the L3-L4 segment under flexion was analyzed in both model 1 and model 4, as shown in Figures 9A, B. The maximum pressure was observed at the anterior portion of the intervertebral disc in both models, but it was 9.8% higher in model 4 than in model 1 (Figure 9C). The highest pressure was observed in the L2-L3, L3-L4, and L4-L5 segments during flexion, as compared to extension, bending, and torque. Among all four models, the pressure in L3-L4 showed the greatest increase during flexion, rising from 1.49 MPa in model 1 and 2 to 1.52 MPa in model 3 and 1.56 MPa in model 4. Unilateral laminotomy resulted in less alteration of spinal biomechanics due to its focused nature, while complete laminectomy and facetectomy could lead to more significant changes, potentially impacting load distribution.

### Maximum shear stress

**FIGURE 10:**
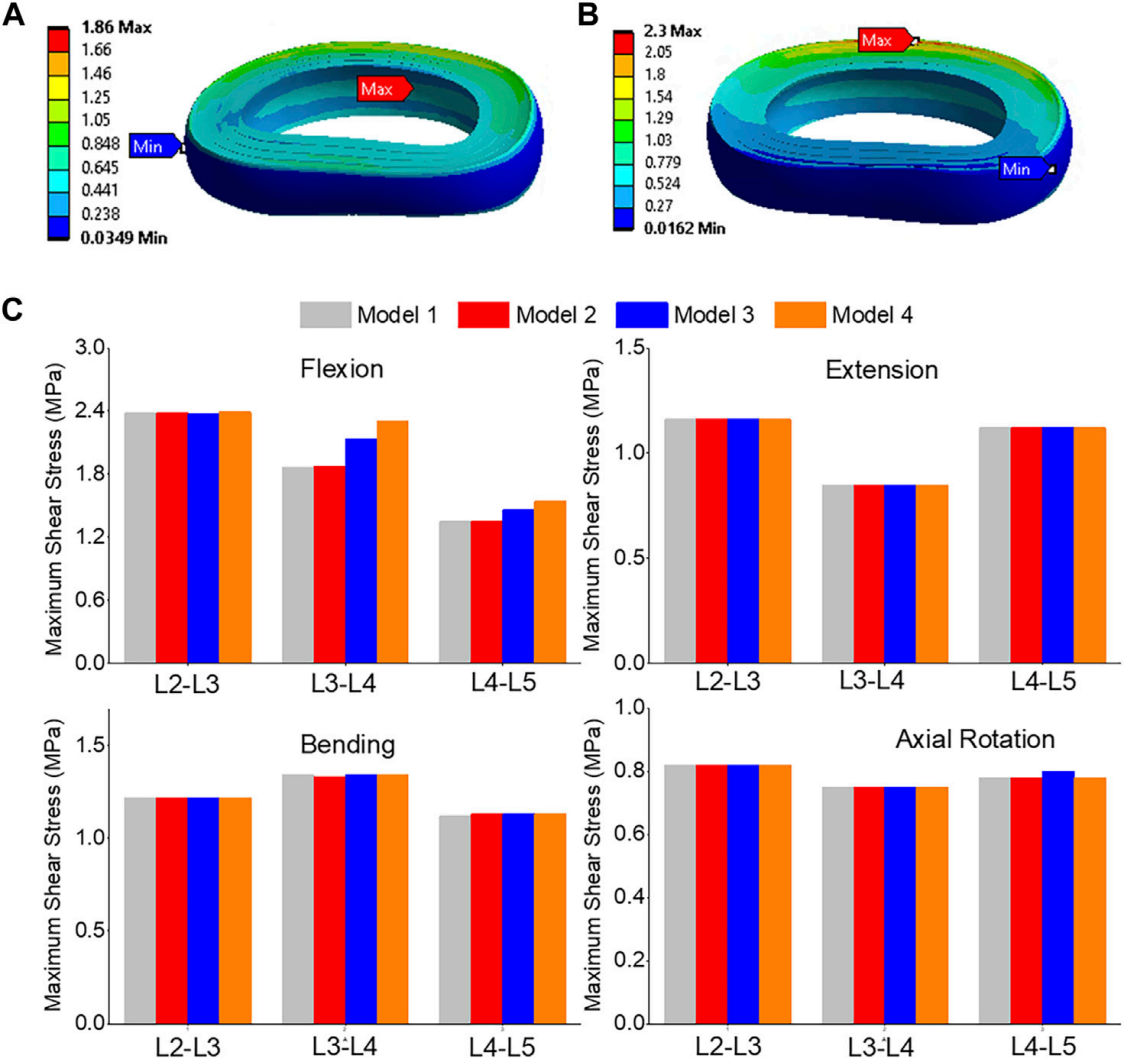
**(A)** A typical shear stress contour of the L3-L4 under flexion for model 1; **(B)** A typical shear stress contour of the L3-L4 under flexion for model 4; **(C)** Comparison of annulus maximum shear stress of Flexion, Extension, Lateral Bending, and Axial Rotation in all four models.

**FIGURE 11:**
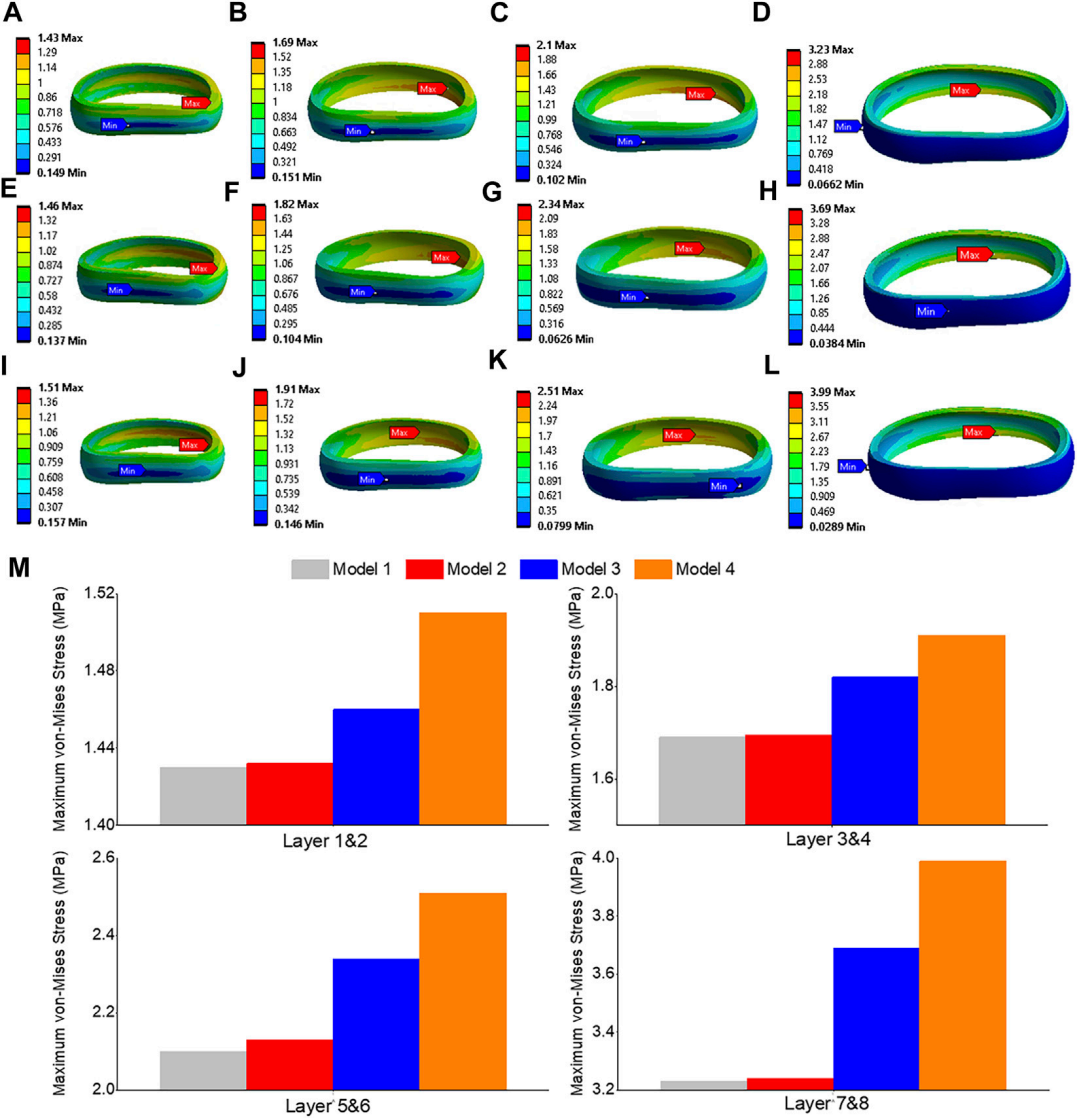
Maximum shear stress contour of the L3-L4 under flexion situation: **(A)** layer 1&2 in model 1; **(B)** layer 3&4 in model 1; **(C)** layer 5&6 in model 1; **(D)** layer 7&8 in model 1; **(E)** layer 1&2 in model 3; **(F)** layer 3&4 in model 3; **(G)** layer 5&6 in model 3; **(H)** layer 7&8 in model 3; **(I)** layer 1&2 in model 4; **(J)** layer 3&4 in model 4; **(K)** layer 5&6 in model 4; **(L)** layer 7&8 in model 4; **(M)** Comparison of annulus maximum shear stress of all layers for all four models.

The highest shear stress was located at the front of the intervertebral disc in both models, but it was 23.6% higher in model 4 than in model 1 (Figures 10A, B). During flexion, the L2-L3, L3-L4, and L4-L5 segments showed the highest shear stress when compared to extension, bending, and torque (Figure 10C). Among all four models, the maximum shear stress in L3-L4 displayed the greatest increase during flexion, increasing from 1.86 MPa in models 1 and 2 to 2.13 MPa in model 3 and 2.3 MPa in model 4. Across all layers, an increase in the maximum shear stress was observed from model 1&2 to model 3 and model 4, with the highest rate of increase observed in layer 7&8 (Figure 11). Complete laminectomy provides substantial decompression of the neural elements but may compromise the posterior tension band, potentially leading to higher shear stress in the absence of sufficient ligamentous support. The degree of facetectomy can vary, ranging from partial to complete removal of the joint. Facetectomy significantly affects the load-bearing capacity of the spine.

### Maximum von-Mises stress

**FIGURE 12:**
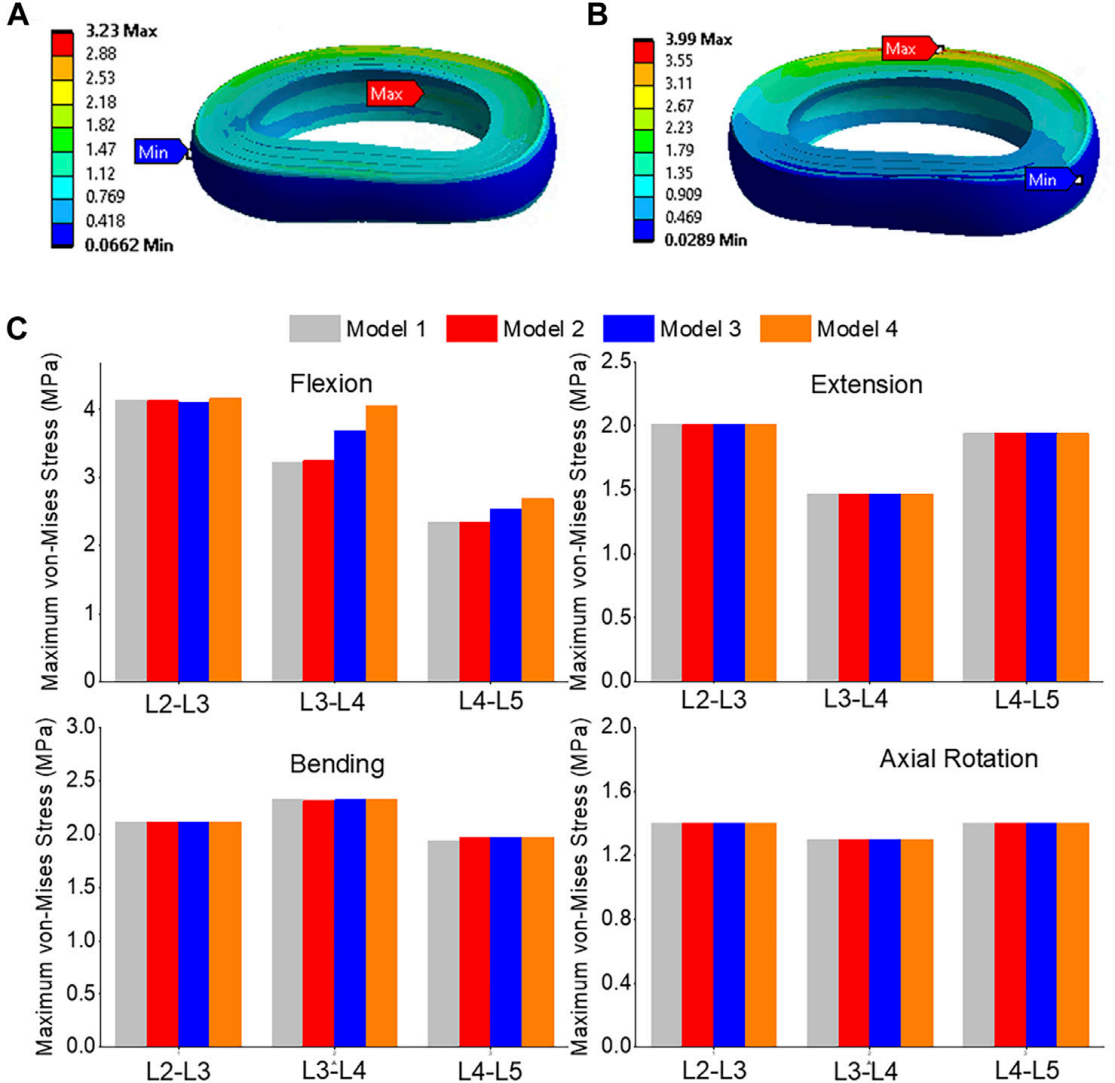
**(A)** A typical von-Mises stress contour of the L3-L4 under flexion for model 1; **(B)** A typical von-Mises stress contour of the L3-L4 under flexion for model 4; **(C)** Comparison of annulus maximum von-Mises stress (top to bottom) of Flexion, Extension, Lateral Bending, and Axial Rotation in all four models.

**FIGURE 13:**
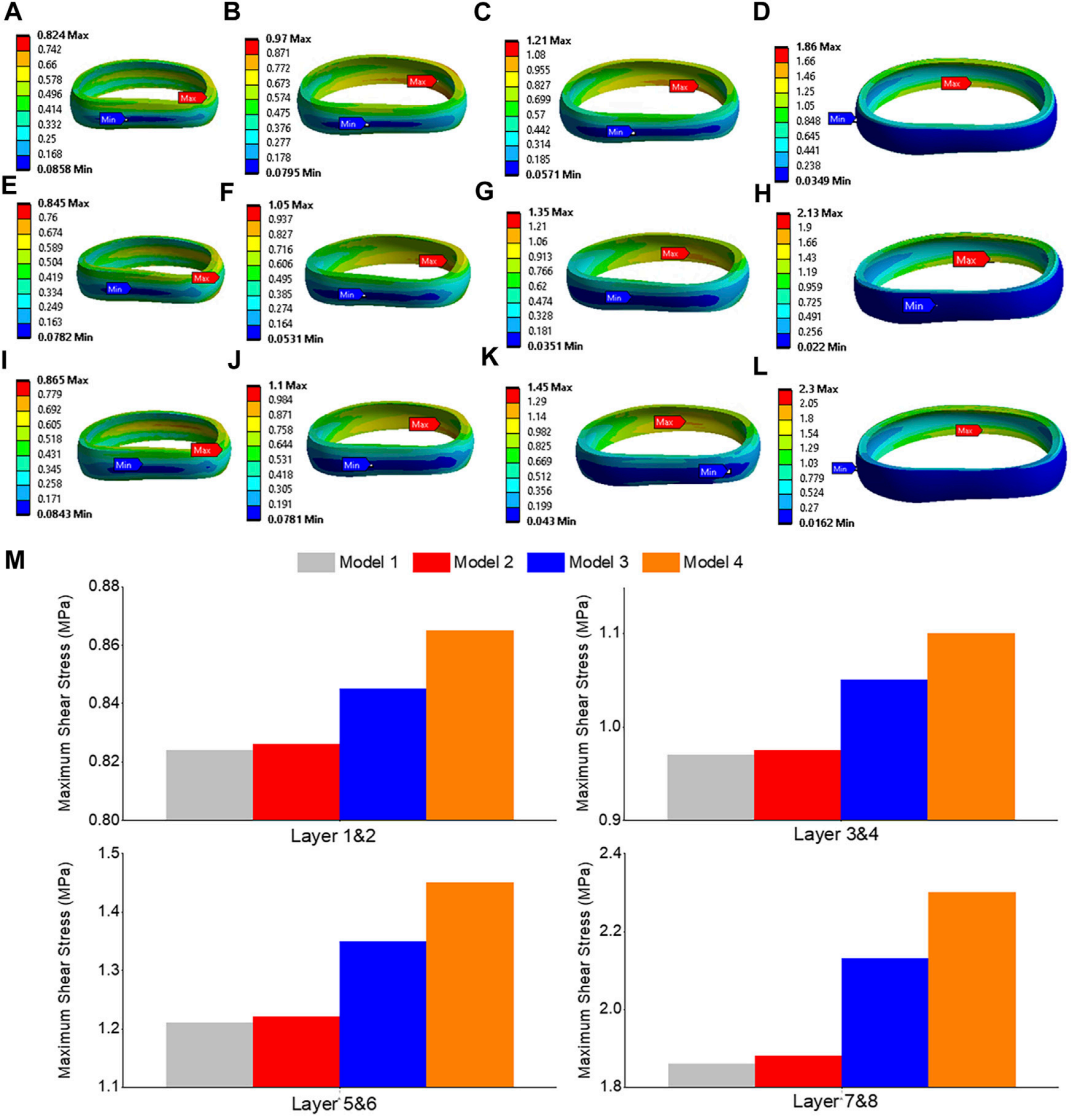
von-Mises stress contour of the L3-L4 under flexion situation: **(A)** layer 1&2 in model 1; **(B)** layer 3&4 in model 1; **(C)** layer 5&6 in model 1; **(D)** layer 7&8 in model 1; **(E)** layer 1&2 in model 3; **(F)** layer 3&4 in model 3; **(G)** layer 5&6 in model 3; **(H)** layer 7&8 in model 3; **(I)** layer 1&2 in model 4; **(J)** layer 3&4 in model 4; **(K)** layer 5&6 in model 4; **(L)** layer 7&8 in model 4; **(M)** Comparison of annulus maximum von-Mises stress of all layers for all four models.

The front of the intervertebral disc in both models had the highest von-Mises stress, with a 23.5% increase in model 4 compared to model 1 (Figures 12A, B). During flexion, the L2-L3, L3-L4, and L4-L5 segments showed the highest von-Mises stress when compared to extension, bending, and torque. Among all four models, the maximum von-Mises stress in L3-L4 showed the greatest increase during flexion, rising from 3.25 MPa in models 1 and 2 to 3.69 MPa in model 3 and 4.05 MPa in model 4 (Figure 12C). In all layers, there was an increase in the maximum shear stress from model 1&2 to model 3 and model 4, with the highest rate of increase observed in layer 7&8 (Figure 13). Overall, the results suggest that during flexion, the L3-L4 segment experiences the highest levels of shear and von-Mises stresses, with model 4 showing the highest levels. This information could be valuable for the design and development of interventions aimed at preventing or treating injuries to the spinal column caused by repetitive bending motions or other activities that place significant stress on this region of the spine.

## Discussion

In our study, we employed a comprehensive loading approach to the spine, incorporating different moments and follower preload values to validate previous finite element analysis (FEA) and clinical research findings. The results indicated that unilateral laminotomy had minimal impact on range of motion (ROM) and pressure. This suggests that this procedure could potentially offer a surgical option with limited biomechanical consequences, while still addressing lumbar spine stenosis. Complete laminectomy resulted in increased spinal flexibility and altered load distribution across vertebral segments due to extensive posterior element removal. This finding underscores the biomechanical implications of this procedure, which might influence the choice of surgical technique. Facetectomy significantly affected spine stability and load-bearing capacity in all flexion situations. This implies that this procedure could potentially compromise spinal integrity, emphasizing the need for careful consideration when opting for facetectomy. Prior biomechanical investigations have generally acknowledged that extensive posterior element removal, such as complete laminectomy, can lead to increased spinal mobility. This aligns with the findings of our study, which showcased elevated ROM in cases of complete laminectomy. Furthermore, the effects of different surgical techniques on load distribution, as highlighted in this study, resonate with existing literature that emphasizes the importance of preserving vertebral stability through techniques that maintain the posterior elements. The study’s findings also echo prior research suggesting that unilateral laminotomy might have relatively minor biomechanical impacts. This consistency underscores the viability of this procedure for certain cases of lumbar spine stenosis, where preserving spinal stability is a consideration.

To our knowledge, we are the first to introduce buffer springs and preload spring to assume the follower load, which helps more accurately simulate the real-world mechanical conditions experienced by the spine during various activities. This spring was attached to the upper surface of the L2 endplate and connected to a floating rigid body, which simulated muscle power using the “joint” command. Several authors have previously conducted finite element analyses on the lumbar spine, focusing on the functional spinal unit or specific levels (Goel et al., 1993; Pawlikowski et al., 2015). Our model exhibited consistent intradiscal pressure results with those of previous studies, such as Xu’s flexion and extension intradiscal pressures (Xu et al., 2017). Other researchers, like Eberlein et al. (2004), validated their models by considering disc degeneration and comparing computational models with experimental data from cadavers. Furthermore, studies by Dreischarf et al. (2014) generated L1-L5 models under various loading conditions, and our model’s range of motion (ROM) and intradiscal pressure results aligned with their findings in intact models (Rohlmann et al., 2009).

From a biomechanical perspective, von-Mises stress is an important measure in the field of mechanics and engineering to assess the stress distribution and potential failure of materials under complex loading conditions (Yao et al., 2006). In the context of the lumbar spine, the von Mises stress is a critical parameter for evaluating the mechanical behavior of the spinal components, such as the annulus and nucleus (Calvo-Echenique et al., 2018). It helps in understanding the stress distribution and potential failure regions within the spinal structure. In our study conducted on the lumbar spine, the von Mises stress was assessed at different levels (L3-L4 and L4-L5) using finite element models. The results showed the maximum von Mises stress values for each model during flexion. Specifically, the maximum von Mises stress at the L3-L4 level increased from 3.25 MPa in models 1 and 2 to 3.69 MPa in model 3 and 4.05 MPa in model 4. Similarly, at the L4-L5 level, the maximum von Mises stress rose from 2.34 MPa in models 1 and 2 to 2.59 MPa in model 3 and 2.69 MPa in model 4. These findings provide valuable insights into the stress distribution patterns and indicate the regions that are subjected to higher stress levels during flexion in the lumbar spine after removing the ligaments and posterior element. By analyzing the von Mises stress values, researchers and clinicians can assess the mechanical behavior of the lumbar spine under different loading conditions, identify areas of potential concern, and make informed decisions regarding surgical interventions or treatment approaches.

Maximum shear stress is another important mechanical parameter used to evaluate the behavior of materials and structures under load (Miccoli et al., 2015). It represents the force parallel to the surface of a material, causing deformation or sliding along the surface. In the study conducted on the lumbar spine, the shear stress was assessed at different levels (L3-L4 and L4-L5) using finite element models. The results indicated the maximum shear stress values for each model during flexion. Specifically, the shear stress increased from 1.86 MPa in models 1 and 2 to 2.13 MPa in model 3 and 2.3 MPa in model 4. Moreover, the maximum shear stress at the L4-L5 level increased from 1.35 MPa in models 1 and 2 to 1.46 MPa in model 3 and 1.54 MPa in model 4. These findings provide insights into the distribution and magnitude of maximum shear stress within the lumbar spine during flexion. Excessive shear stress can potentially contribute to disc degeneration and related spinal conditions. By analyzing the shear stress values, researchers and clinicians can assess the structural integrity and potential areas of concern, as regions susceptible to shear stress related damage or failure. Understanding the shear stress response helps in designing appropriate treatment strategies, surgical interventions, or interventions aimed at reducing excessive shear forces and minimizing the risk of associated complications.

In this study, we aimed to improve the accuracy of lumbar spine biomechanical simulations by considering the layered structure of the annulus fibrosus and accounting for variations in fiber orientation and stiffness across its different lamellae. To achieve this, we added four concentric rings, each 1.5 mm thick, around the nucleus pulposus to form the annulus fibrosus. Additionally, we embedded eight layers of fibers within the annulus. The fibers in the outermost layer have the highest elastic strength, and each concentric layer decreases by a scalar factor as it reaches the innermost layer. The nonlinear fiber stress strain curves of the innermost layers (1 & 2) to the outermost layers (7 & 8) were incorporated in the model. Several prior studies have incorporated layered annulus models (Schmidt et al., 2006). By utilizing these advanced modeling techniques, we aimed to better represent the complex behavior of the annulus fibrosus to compare with the previous biomechanical models, which often simplify the annulus fibrosus by assuming it to be a homogeneous and isotropic material (Ye et al., 2022). These studies have shown that the annulus fibrosus exhibits significant regional variations in composition, fiber orientation, and mechanical properties (Acaroglu et al., 1995). These variations can influence the distribution of stress and patterns of deformation within the annulus fibrosus under different loading conditions. In our study, we observed variations in von Mises stress and maximum shear stress contours within each layer. These findings provide a detailed comparison and valuable guidance regarding potential areas of concern in surgery. Overall, our study highlights the importance of considering the complex nature of the annulus fibrosus in biomechanical modeling of the lumbar spine. By incorporating more realistic anatomical and material properties, we can enhance the accuracy of simulations and gain deeper insights into the biomechanical behavior of the lumbar spine.

The outcomes of this study hold practical implications for clinical practice and biomechanical research. The insights gained from comparing different surgical techniques and their effects on lumbar spine biomechanics can inform surgical decision-making. Surgeons can consider these findings when selecting the appropriate procedure for treating patients with lumbar spine stenosis, aiming to achieve optimal outcomes while minimizing potential risks. Additionally, the study contributes to the broader field of biomechanics by enhancing the understanding of how specific surgical interventions impact the mechanical behavior of the lumbar spine. This knowledge can guide the development of more effective surgical techniques and rehabilitation strategies, ultimately improving patient outcomes and quality of life. Furthermore, the study highlights the significance of accounting for complex factors like posterior element removal and ligament alterations in biomechanical modeling, offering valuable insights for researchers working on similar studies or modeling endeavors.

There are several limitations in this study. While employing the intact model geometry for the stenosis models might not comprehensively encompass anatomical variations due to stenosis progression, it is important to emphasize that the study primarily centered on assessing the biomechanical consequences of surgical resections. It is worth noting that the absence of supplementary instrumentation was intentional, with the aim of specifically isolating the biomechanical repercussions arising from the graded laminotomy techniques. The anatomy of the vertebrae is intricate and can differ between anatomical locations and patients (Panjabi et al., 1992), making comparisons with other studies difficult. To account for this inter-subject variability, multiple models need to be analyzed. Future research efforts should continue to refine and validate models, taking into account the complex structure and properties of the annulus fibrosus to provide a more accurate representation of the biomechanical behavior of the lumbar spine. Advancements in imaging techniques, such as magnetic resonance imaging (MRI), will allow more detailed characterization of the annulus fibrosus at the microstructural level. These imaging techniques enable the extraction of patient-specific data, such as fiber orientation and material properties, which can be incorporated into finite element models for more realistic simulations.

## Conclusion

This study aimed to construct four distinct finite element models in order to compare various biomechanical parameters such as range of motion (ROM), pressure, von Mises stress, and maximum shear stress within the lumbar spine (L2-L5). The first model mirrored the intact lumbar spine and was successfully validated. While unilateral laminotomy had minimal impact on ROM and pressure, complete laminectomy led to heightened spinal flexibility and changed load distribution due to extensive posterior element removal. In contrast, facetectomy significantly influenced spinal stability and load-bearing capacity during all flexion scenarios. The findings emphasized that posterior element and ligament removal, as undertaken in stenosis treatment at L3-L4 and L4-L5 levels, caused increased flexion and axial rotation at the surgical site.

## Data Availability

The original contributions presented in the study are included in the article/Supplementary material, further inquiries can be directed to the corresponding author.
